# A Rare Presentation of Sarcoidosis in a Young Male With Acute Renal Failure: A Case Report and Literature Review

**DOI:** 10.7759/cureus.49512

**Published:** 2023-11-27

**Authors:** Ivonne De la Hoz, Alsayed Osman, Robert Ryad, Weiying Li, Shuva Shah, David Notman, Luis Isea, Daniel Tambunan

**Affiliations:** 1 Internal Medicine, AdventHealth Orlando, Orlando, USA; 2 Internal Medicine, Mountain Air Health Education Center, Asheville, USA

**Keywords:** sarcoidosis, pancytopenia, granulomatous interstitial nephritis, acute renal failure, renal sarcoidosis

## Abstract

Sarcoidosis presents in a variety of ways, but historically, renal involvement has been considered rare with an incidence of 0.7% and is seldom the presenting feature of the illness. Concomitant involvement of kidney and bone marrow is extremely rare. Atypical forms of presentation, such as in this case, may pose a true diagnostic challenge.

A 20-year-old African-American male presented to the emergency department with vague symptoms including fatigue, malaise, anorexia, right-sided lower back pain, and nausea. Acute kidney injury was clearly evident, creatinine was 19.78 mg/dL (normal range 0.60-1.20 mg/dL), and BUN was 124.0 mg/dL (normal range 5.0-25.0 mg/dL). Laboratory results were also remarkable for leukopenia, microcytic anemia, hyperkalemia, anion gap metabolic acidosis, and non-PTH dependent hypercalcemia. Interestingly, urinalysis was equivocal and both chest x-ray (CXR) and abdominopelvic computed tomography (CT) scan were unrevealing. The patient was admitted to the hospital and required renal replacement therapy to stabilize his clinical condition while planning for a renal biopsy that was later performed. While awaiting pathological results, pancytopenia developed, and a bone marrow biopsy was then obtained. On further investigation, angiotensin-converting enzyme (ACE) turned out to be significantly elevated suggesting sarcoidosis. Renal biopsy showed moderate acute tubular injury, tubulitis, extensive interstitial edema, and infiltration by numerous non-caseating granulomas, which confirmed the diagnosis of sarcoidosis. Bone marrow histopathology revealed hypocellularity but no granulomatous infiltration. The patient remained largely asymptomatic throughout his hospital stay, with no signs or symptoms suggesting the involvement of other organs. High-dose corticosteroids were started and continued outpatient after discharge while still on hemodialysis. Pancytopenia resolved while on glucocorticoids and improvement in renal function was such that after roughly two months of steroids, renal replacement therapy was no longer necessary.

Overall, kidney injury severe enough to require hemodialysis associated with pancytopenia in a previously healthy 20-year-old constitutes a rather rare sarcoidosis presentation. This highlights the importance of considering sarcoidosis as a possible cause of kidney and bone marrow dysfunction and emphasizes the need for timely biopsy to facilitate accurate diagnosis and early initiation of appropriate therapy to avoid delayed or inadequate care, especially considering that even severe damage is potentially reversible when identified early and treated promptly.

## Introduction

Sarcoidosis is a multisystem inflammatory condition that can affect any organ therefore presentation varies largely. It has been estimated that the prevalence of Sarcoidosis stands at about 77/100,000 individuals, with variations among different geographic regions, and the most common incidence occurring in the fourth decade of life [1].

Etiology remains misunderstood, but genetic predisposition and even environmental factors are believed to play a role. Multiple human leukocyte antigen (HLA) genes are involved, and certain HLA variants have been associated with specific organ involvement and severity of disease. Familial clusters exist that support genetic mutations. HLA proteins are responsible for presenting antigens to CD4 cells. This interaction will lead to the activation of cytokines and inflammatory markers, and ultimately an aberrant immune response where macrophages end up forming granulomas. Regarding environmental factors, workers exposed to silica, and insecticides, like firefighters and farmers have shown increased incidence. Certain microorganisms, such as Propionibacterium and Mycobacterium, have also been linked, but the connection is unclear. Vimentin, a structural protein, may also play a role [2,3].

Definitive diagnosis is through histopathologic confirmation after biopsy, which typically will be obtained from the most accessible organ involved, preferably lymph nodes or skin. Infiltration with non-caseating granulomas represents the hallmark of the disease and many times will lead to organ architecture distortion [2].

Over 90% of those with sarcoidosis have lung or lymph node involvement within the thoracic cavity, usually causing cough and dyspnea. Skin involvement can be seen in around 30% of cases. Lupus pernio is the most classic presentation, with violaceous plaques covering the nose, cheeks, and ear lobes. The prevalence of ocular sarcoid ranges from 10% to 50%. The most common sign is uveitis, which is usually anterior and bilateral [3,4].

Rarely, sarcoidosis can cause kidney involvement, which is most often due to disordered calcium breakdown yielding renal calcium deposits, however, inflammatory infiltration can also lead to kidney dysfunction. In the vast majority of cases, this is preceded by extrarenal symptoms [4]. Sarcoidosis can cause hematologic abnormalities, such as anemia, leukopenia, and thrombocytopenia, but these are infrequent, and the presence of pancytopenia is even rare [5]. The following depicts a case of a 20-year-old African American male who initially presented with acute renal failure secondary to sarcoidosis and developed pancytopenia.

## Case presentation

A 20-year-old African American male patient presented to the Emergency Department with complaints of right para-spinal back pain and supra-pubic abdominal pain of one-week duration accompanied by progressive fatigue, lethargy, generalized weakness, and nausea. In addition, the patient reported decreased appetite and unintentional weight loss of at least 5 lbs. over a duration of two to four weeks, decreased urinary output, and constipation. He reported a history of Idiopathic Thrombocytopenic purpura (ITP) four years prior, which was treated with complete resolution. The patient was allergic to non-steroidal anti-inflammatory drugs but did not have any relevant family, surgical, or social history. He denied any illicit drug use, herbal supplements use, or home medications. Upon ED arrival, the patient was afebrile, normotensive, and only slightly tachycardic. He was underweight with a body mass index (BMI) of 15.77 kg/m^2^ (height 1.77 m and weight 49.9 kg). The physical exam was only remarkable for mild suprapubic and right costovertebral angle tenderness. There was very mild abdominal distension, but no signs of edema anywhere. Further investigations revealed a significantly elevated creatinine (19.78mg/Ll) and BUN (124.0mg/dL). He was also hyperkalemic (5.9mmol/L), and with an anion-gap metabolic acidosis (Table 1). These findings indicated severe kidney dysfunction and uremia, which provided a plausible explanation for his initial non-specific symptoms. Creatine kinase was not elevated, and the urinalysis revealed microscopic hematuria, non-nephrotic range proteinuria, and occasional calcium oxalate crystals.

**Table 1:** Basic metabolic panel relevant results on admission.

Component	Day 0
Sodium (135-145 mmol/L)	138
Potassium (3.5-5.0 mmol/L)	5.9
Chloride (98-110 mmol/L)	102
Bicarbonate (22.0-29.0 mmol/L)	18
Anion Gap (5-15 mmol/L)	18
BUN (5.0-25.0 mg/dL)	124
Creatinine (0.60-1.20 mg/dL)	19.78
EGFR (>90 mL/min/1.73 m^2^)	3.1
Calcium (8.5-10.5 mg/dL)	12.4

Non-PTH dependent hypercalcemia was noted upon admission with a moderately elevated corrected calcium level (12.4mg/dL) and decreased PTH. In addition, unexplained leukopenia was noted (2.65 × 10^3^/µL) and microcytic hypochromic anemia (hemoglobin 11.8 × 10^3^/µL, MCV 75.4 fL, MCH 23.6 pg). On the other hand, the platelet count was normal (222 × 10^3^/µL). Subsequently, iron, ferritin, iron saturation, transferrin, and TIBC were evaluated and found to be all within the normal range.

A chest x-ray (CXR) did not show any relevant abnormality. In like manner, a computed tomography (CT) of the abdomen and pelvis did not show any significant findings, other than an incidentally discovered nonobstructive renal calculus. There were no kidney masses or hydronephrosis seen. Non-specific findings including diffuse mesenteric edema, trace ascites, and increased liver attenuation were reported.

At this point, there were many unanswered questions, beyond any doubt there was a 20-year-old with kidney failure, seemly acute, however, an underlying chronic and more insidious process could not be ruled out. The only past medical records available were from four years ago, which showed normal creatinine. Since the patient had not been seen by any healthcare provider for the last four years, the precise time the kidney function began to deteriorate remains a mystery. Initially, electrolyte disturbances were corrected while arranging for hemodialysis which was started afterward.

Since the workup so far had been inconclusive, the differential diagnosis at this point was broad, and the etiology was unclear. The clinical picture was not typical for nephritic or nephrotic syndrome, and additional tests including complement, ASO titers, PPD, HIV, ANA, rheumatoid factor, C-ANCA, and P-ANCA, were unremarkable. Clinical condition improved with dialysis, and after optimization, a kidney biopsy was performed. While awaiting kidney biopsy results, the platelet count that initially had been within normal range dropped significantly, and the patient developed pancytopenia (Table 2). A peripheral blood smear was performed but did not show any relevant findings, a biopsy of the bone marrow was later obtained.

**Table 2:** Complete Blood Count relevant results on admission compared to hospital day 7 when pancytopenia became more notorious.

	Day 0	Day 7
WBC (4.40 - 10.50 10*3/uL)	2.65	1.43
Hemoglobin (12.6 - 16.7 g/dL)	11.8	9.7
Platelet (139 - 361 10*3/uL)	222	74

In the meantime, the angiotensin-converting enzyme turned out to be significantly elevated, which in conjunction with hypercalcemia, pointed out to Sarcoidosis as the underlying cause of kidney failure in this patient. Therefore, high-dose IV steroids were initiated empirically.

**Figure 1:**
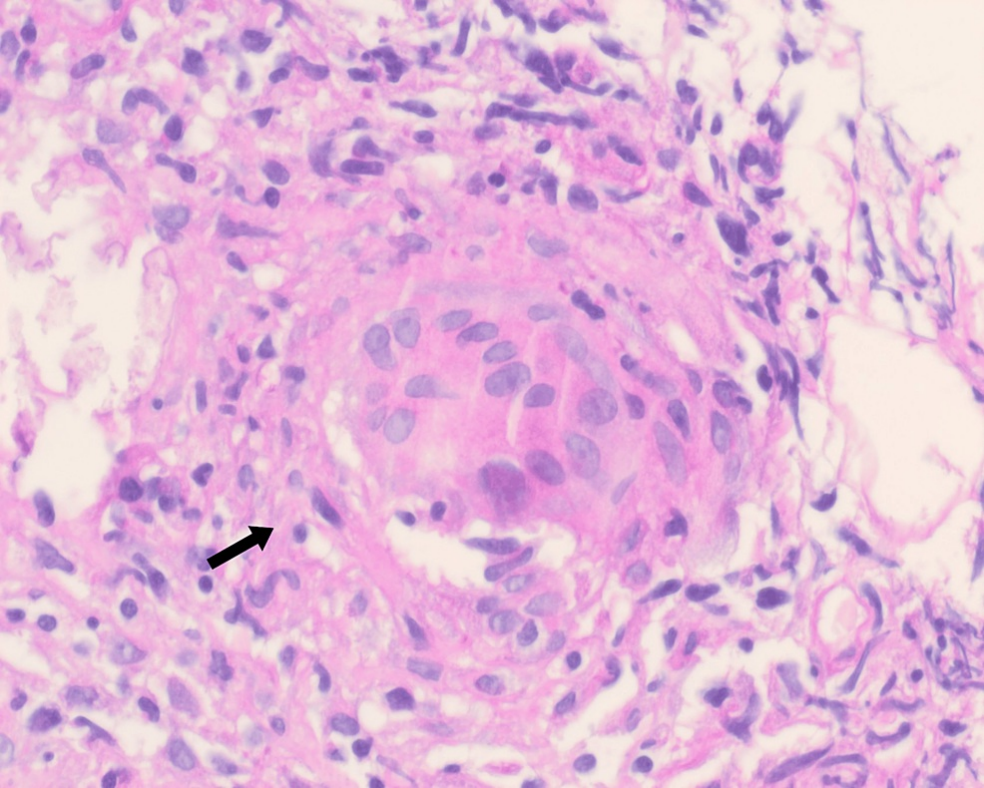
Kidney biopsy with hematoxylin and eosin stain, note non-caseating granuloma (arrow).

Kidney biopsy revealed moderate acute tubular injury, tubulitis, extensive interstitial edema, mild mesangial C3 deposition, and of greater significance, infiltration by numerous non-caseating granulomas, the widely known sarcoidosis hallmark (Figure 1).

Since pulmonary involvement is the most frequent form of presentation for sarcoidosis yet chest imaging had been unremarkable thus far, a high-resolution chest CT scan was requested as part of the sarcoidosis workup to more accurately characterize lung and hilar lymph node structures. The goal was to confirm pulmonary involvement, despite the absence of respiratory symptoms, or to rule it out. Interestingly, no lymphadenopathy nor any signs of sarcoidosis were demonstrated.

The results of the bone marrow biopsy were inconclusive; they only revealed hypocellularity and no other abnormalities, such as infiltrative processes. While on steroids, the platelet count began to rise and then became stable. The patient underwent a thorough workup to identify the underlying cause of pancytopenia. It was impossible to confirm sarcoid as the culprit but there was an excellent response to glucocorticoids.

The patient remained largely asymptomatic throughout his hospital stay, with no signs or symptoms suggesting the involvement of other organs. Still, this does not completely rule out the possibility of mild or early damage to other organs.

The patient was discharged and continued frequent close follow-up with nephrology while on outpatient hemodialysis. Surprisingly, after about two months of high-dose steroids (prednisone 60 mg daily), renal function improved significantly to the point where this young man no longer required renal replacement therapy.

## Discussion

Sarcoidosis is often suspected and diagnosed in patients with pulmonary symptoms or incidental hilar adenopathy on chest imaging. An isolated kidney injury, severe enough to require hemodialysis in a previously healthy 20-year-old, constitutes a rather rare sarcoidosis presentation. Historically, renal involvement is considered infrequent in sarcoidosis with an incidence of 0.7% and is seldom the presenting feature of the illness. However, recent evidence suggests that renal involvement in sarcoidosis might be underestimated. A retrospective study reports that 32% of 78 patients with sarcoidosis were diagnosed with renal failure [6]. Another multi-center cohort study showed that the kidney was involved in 12% of sarcoidosis cases. Dysregulated calcium metabolism is commonly seen in sarcoidosis, and it often manifests as nephrolithiasis and nephrocalcinosis secondary to hypercalcemia, which rarely leads to clinically significant kidney disease. The histopathological hallmark of renal sarcoidosis is granulomatous interstitial nephritis (GIN), which accounts for 30% to 78.7% of renal sarcoidosis [7-9]. Atypical histological findings such as glomerulonephritis [10], amyloidosis [11], and vasculitis [12] are occasionally reported, however, causality and mechanism have not been proven [13]. As a matter of fact, some atypical clinical features bring up concerns about possible confounding factors in the diagnosis of sarcoidosis with other granuloma-causing etiologies such as tuberculosis, fungal infection (Histoplasma, Candida, Rhodococcus) and drug-induced interstitial nephritis (NSAIDs, PPI) [14,15]. 

It is worth noting that even though noncaseating granuloma is the classic histological feature of renal sarcoidosis, this feature alone cannot establish the diagnosis of sarcoidosis due to its non-specific nature. An analysis of 46 cases with GIN revealed that 28.9% of cases resulted from renal sarcoidosis, while 44.7% were drug-induced [16]. Another retrospective study shows among 40 cases with renal granulomatosis, 50% were caused by sarcoidosis; other etiologies include granulomatosis with polyangiitis (GPA), Wegener’s granulomatosis, foreign body giant cell reaction, mycobacterium avium, and Crohn’s disease [17]. Therefore, it is fundamental to understand that renal sarcoidosis is a diagnosis of exclusion. In our case, kidney biopsy showed classic noncaseating granulomatous interstitial nephritis and there was a response to steroids. A negative history of drug usage and workup excluding infection, vasculitis, and autoimmune causes established the clinical-pathological diagnosis of renal sarcoidosis. Most other case reports on renal sarcoidosis present with a more insidious onset, less debilitating clinical progression, and did not require immediate renal replacement therapy [15,18,19]; in significant contrast, the clinical course of this young male featured acute kidney failure that required hemodialysis, and it was the first manifestation that prompted the work up and led to the diagnosis of sarcoidosis. Furthermore, sarcoidosis is known as a systemic granulomatous disease involving the lungs in 90% of cases [20]. Interestingly, high-resolution chest CT did not find any evidence of hilar adenopathy or pulmonary sarcoidosis in this patient. Follow-up on lung manifestation will reveal if isolated renal sarcoidosis precludes the occurrence of pulmonary sarcoidosis in certain cases. Last but not least, this is a unique case because acute kidney failure was complicated with pancytopenia in one who had past medical history of ITP. Concomitant involvement of kidney and bone marrow is extremely rare: only two cases have been reported. In first case report, an elderly male presented with pancytopenia and was found to have kidney injury during routine lab work, then sarcoidosis was confirmed by lung biopsy and bone marrow biopsy which showed granuloma, but kidney biopsy was not obtained. In another case, the diagnosis of sarcoidosis was also established by bone marrow biopsy which showed granuloma without evidence of renal pathology [21]. The majority of case series with isolated bone marrow involvement, pancytopenia was exclusively caused by granulomatous infiltration in bone marrow [22].

It is noteworthy that bone marrow biopsy in our case showed hypoplasia due to loss of hematopoietic stem cells with no evidence of granuloma infiltration, which pointed to a distinct pathogenetic process compared with previous reported cases. This is the first case reported with acute kidney failure requiring renal replacement therapy as the predominant feature at initial presentation complicating with pancytopenia resulting from hypocellularity rather than bone marrow sarcoid infiltration. The association between sarcoidosis and pancytopenia in the setting of renal sarcoidosis has rarely been investigated. In comparison, thrombocytopenia is a relatively common type of hematological manifestation, which ties with the history of ITP in this patient. The observation that sarcoidosis and thrombocytopenia occur together frequently makes it difficult to argue against a correlation, and strongly points towards a specific immunogenetic predisposition. The studies on thrombocytopenia complicated with sarcoidosis might shed some light on the pathogenesis of pancytopenia in the setting of sarcoidosis. A retrospective analysis of 31 cases of sarcoidosis with isolated thrombocytopenia revealed only four cases showed granuloma in bone marrow, whereas it was suspected that the likely etiology in other cases was autoimmune thrombocytopenia purpura [23]. Based on observation and literature review, there is likely more than one pathogenetic process resulting in pancytopenia [24]. We propose four putative mechanisms to explain pancytopenia in the setting of renal sarcoidosis. First, bone marrow replacement by sarcoid infiltration can disrupt hematopoietic cell proliferation and lead to pancytopenia, as it is the case in the majority of reports. Second, as it is widely accepted that sarcoidosis develops in genetically predisposed hosts through a cell-mediated immune response to unidentified antigens [25], concurrent hypergammaglobulinemia secondary to generalized B-cell hyperactivity poses the risk of cross-reacting with self-antigen on hematopoietic cells and resulting in autoantibody mediated cellular destruction, which manifests as hypoplasia/aplasia histologically [26]. Furthermore, massive production of cytokines by abnormally activated T-lymphocytes in the pathological process of sarcoidosis can lead to bone marrow suppression [27]. A myriad of cytokines such as interleukin 2 (IL-2) and interferon γ (IFN-γ), tumor necrosis factor (TNF), IL-12, and IL-18 are increased in sarcoidosis due to immuno-activation, while IL-2, IFN-γ and TNF-α are known as negative regulators of stem and precursor cell proliferation and survival. Therefore, massive production of cytokine in immune over-activation remains as a plausible pathway resulting in bone marrow suppression. Lastly, hypersplenism can lead to pancytopenia in sarcoidosis [28]. The presence of splenomegaly due to direct splenic involvement or due to the liver involvement with subsequent portal hypertension, and hypodense splenic nodules are few imaging findings suggesting hypersplenism as a cause of pancytopenia. Improved cell count post splenectomy also supports this mechanism [29]. Abdominal imaging in our case, however, did not reveal splenic involvement.

Notably, Meshram et al. presented a case of a nine-year-old female child who had multiple nodular skin lesions on her hands and feet, paleness, and recurrent episodes of epistaxis since she was three years old. The initial assessment had shown pancytopenia, and at some point, the child underwent bone marrow biopsy, but the results did not reveal granulomatous infiltration, as in our case. However, symptoms persisted, and at the age of nine, a repeated marrow biopsy did reveal several non-necrotizing granulomas, leading to the diagnosis of sarcoid [30]. Therefore, the absence of infiltration currently is insufficient to rule out sarcoidosis as the underlying cause of pancytopenia in this young male.

Nevertheless, further research is warranted to delineate the underlying mechanism of pancytopenia in the setting of renal sarcoidosis [31]. Corticosteroids are the treatment of choice for renal sarcoidosis, treatment dosing and duration depends on the severity and fibrosis and tubulointerstitial nephritis on kidney biopsy. An early response to steroid seems to be the key prognostic factor for renal survival [7]. Other agents such as methotrexate, azathioprine, hydroxychloroquine, and mycophenolate can be considered among patients who cannot tolerate or respond to glucocorticoids; however, the effects of these agents on renal sarcoidosis are uncertain due to a lack of data [32].

## Conclusions

In conclusion, we report a rare case of sarcoidosis with a unique constellation of acute kidney failure and pancytopenia. Renal sarcoidosis can develop into end-stage kidney disease. Our case emphasizes the need for timely kidney biopsy to enable accurate diagnosis and early initiation of appropriate therapy to halt disease progression, and potentially reverse damage, as it did in this instance.

Based on emerging evidence and observation, we also clarified a common misconception and clinical pitfall about sarcoidosis, which has been traditionally conceived as a pulmonary disease rather than a systemic condition that can affect any organ.
